# Edinburgh Surgical Hospital

**Published:** 1830-08

**Authors:** 


### EDINBURGH SURGICAL HOSPITAL.

### *Fungous Tumor of Mamma.**

Jean Hey, æt. thirty-seven, admitted 19th February, on account of a large fungous tumor growing from the upper part of the mamma, not involving the nipple or skin below it, and not seeming to adhere to the subjacent parts. It is of an irregular shape, of a dark-red colour, of a soft consistence, and bleeds freely when touched; the discharge from it is thin, dark coloured, fœtid, and very copious. The patient is extremely emaciated, her countenance is anxious, and her complexion of a remarkable unhealthy-looking yellow hue. She has little appetite, and what food she does take is generally rejected by the stomach. She has frequent fits of sickness, which she ascribes to the smell from her breast; she complains much of pain, and passes restless nights. Pulse quick and weak.

Last May she had an infant seven months old at the breast, and was much confined to the house. She caught cold one day from going out, and was attacked with erysipelas in the face, which went off the following morning. She continued to be sick and squeamish occasionally for six weeks, her appetite being bad, and her thirst great, when she felt a small hard lump just under the nipple of her left breast. It increased in size, the child still continuing to suck, and formed a large elastic swelling, discoloured on the surface, and very painful. After poulticing it for some time, she applied to a surgeon, who made an incision, and evacuated twenty ounces of a dark-coloured fœtid fluid, and on two other occasions, within the following ten days, discharged two teacupsful of the same sort of matter. Poultices were again applied, but the pain and discharge continued, and an abscess formed, which being opened in January last, was found to contain six ounces of thick fœtid pus. The opening did not close, but the skin, as she described it, began to fall off in small pieces round the wound, while fungous masses at the same time protruded.

23d. Mr. Syme, being willing to afford the patient the only chance

* From Mr. Syme's Quarterly Report. Edinb. Med. and Surg. Journal.

she had of recovery, proceeded to excise her breast. He included the tumor along with the nipple, within two semilunar incisions; it adhered slightly to the pectoral muscle, and, after dissecting it off, he discovered a small round tumor lying under the muscle, which he likewise removed. The edges of the wound were brought together with some difficulty, owing to the large quantity of skin removed, and retained by means of stitches. She was ordered beef-tea and wine in small and repeated doses. The diseased part being cut through after the operation, exhibited a very characteristic specimen of medullary sarcoma.

24th.—Has passed a very restless night, having had profuse diarrhœa. She was ordered half-grain opium pills, to be taken according to circumstances; the stitches to be supported by broad pieces of plaster; beef-tea and wine continued.

25th.—The diarrhœa ceased after the first pill. She is looking a good deal better; sickness less; pulse stronger; countenance not so anxious.

March 2d.—She is improving rapidly. Her appetite is now pretty good; pulse stronger; wound looking healthy. To have porter.

15th.—Wound almost completely healed; desires to go home to attend to her family.

I have given this case at full length from the journals of the Hospital, both because the history of it is rather unusual, and because it shows the possibility of complete temporary recovery by operation from apparently the most hopeless cases of this disease. When I was asked to see this poor women, she was in the most wretched state it is possible to conceive; the air of the room was poisoned with the stench that proceeded from a discharge so copious as to drench her clothes and the bed on which she lay; her stomach rejected food; and her pulse could hardly be felt. The second time I visited her, I found her busy in the performance of domestic duties, strong and active, while the breast was perfectly healed; and apparently free from any disposition to give her further trouble.* It has long seemed to me that we are in the custom of comprehending under the title of medullary sarcoma, many morbid growths of very different morbid tendency. Some of these tumors never fungate, though allowed to attain a great size; many of them fungate, but never bleed; others manifest the most remarkable hemorrhagic disposition; and there is the greatest variety with respect to their recurrence after removal. Such being the case, it would seem to be our duty, so long as we have not ascertained the distinctive characters, if there be any, of those of a malignant nature, to afford the patient a chance, by performing the operation whenever the whole existing disease can be taken away.

* Since this was written, I have learned that her health is again breaking up.

